# Sex Differences in Stroke in Cameroon: Results From a One‐Year Prospective Follow‐Up Cohort Study

**DOI:** 10.1002/hsr2.70841

**Published:** 2025-05-19

**Authors:** Clovis Nkoke, Ahmadou Musa Jingi, Jean Jacques Noubiap, Cyrille Nkouonlack, Anastase Dzudie

**Affiliations:** ^1^ Faculty of Health Sciences University of Buea Buea Cameroon; ^2^ Faculty of Health Sciences University of Bamenda Bamenda Cameroon; ^3^ Division of Cardiology, Department of Medicine University of California‐San Francisco San Francisco California USA; ^4^ Faculty of Medicine and Biomedical Sciences University of Yaounde 1 Yaounde Cameroon

**Keywords:** Cameroon, outcome, risk factors, sex, stroke

## Abstract

**Background and Aims:**

Data suggests that men and women have different vascular risk profiles, clinical characteristics, and outcome trajectories after stroke. There is a dearth of data on sex differences in stroke in Cameroon. We aimed to examine sex differences in stroke in a 1‐year follow‐up prospective cohort study in Cameroon.

**Methods:**

This prospective cohort study included patients who were hospitalized for acute stroke and who were discharged between January 2013 and December 2013. Patients were followed up for 12 months. We compared differences in risk factors, clinical presentation at baseline, and mortality within 12 months of follow‐up between men and women. A *p* value < 0.05 was considered as threshold for statistical significance.

**Results:**

A total of 254 patients with stroke were included in this study at baseline, including 135 (53.1%) men. Women were significantly older than men (65.7 ± 15.5 years vs. 59.2 ± 12.4, *p* < 0.001). Men were more likely to smoke and consume alcohol when compared to women (all *p* < 0.001). There was no significant difference in stroke severity on admission between men and women according to the National Institute of Health stroke scale (9.8 vs. 11.2, respectively, *p* = 0.137). Women had a significantly higher disability on admission compared to men (modified Rankin Score (mRS) = 3.9 vs. mRS = 3.7 respectively, *p* < 0.001). There was no significant difference in stroke subtype between men and women. At the end of the 1 year follow up, there was no statistically significant difference in disability (*p* = 0.22) and the 1‐year mortality (*p* = 0.329) between men and women.

**Conclusions:**

Men with stroke were younger and were more likely to smoke and consume alcohol. Women had greater stroke disability on admission. There was no significant difference in disability and in 1‐year mortality between men and women, although mortality was higher in women.

## 1 Background

Stroke is a major public health problem globally. It is the leading cause of death and disability for both men and women worldwide [1, 2]. Sub‐Saharan Africa seems to have the highest incidence, prevalence, and case fatality rates of stroke compared to other geographic regions [3, 4, 5].

Sex differences in stroke have been examined in many countries, but results from these studies are inconsistent, with most of these reports coming from Europe, America, and Asia [6, 7, 8, 9, 10, 11, 12, 13, 14]. Some studies showed that stroke affects men more than women, but women had poorer outcomes [6, 7, 8, 9]. However, some studies reported no sex differences in survival rate [10, 11], and others have shown that women had better outcomes than men after stroke [12, 13]. Even though stroke affects men and women, there are more strokes and stroke deaths in women than men, which can be explained in part by women's longer life expectancy, differences in clinical presentation, stroke care, and outcomes [2, 15, 16]. After stroke, functional outcomes and quality of life (Barthel index) are worse in women compared to men after adjustment for baseline differences in age, prestroke function, and comorbidities [15]. Various covariates might independently or together help to explain gender differences in stroke [15].

There is a dearth of data on sex differences in stroke in sub‐Saharan Africa, where the burden of stroke is greatest. A previous study from Cameroon demonstrated no sex differences in terms of stroke severity, length of hospital stay, case fatality, and functional outcome on admission [14]. However, this study did not examine sex differences prospectively. To the best of our knowledge, no study has examined the sex differences in stroke in a prospective follow‐up study in Cameroon. The aim of this study was to examine sex differences in age, stroke risk factors, clinical presentation, severity, and outcomes in a 1‐year follow‐up prospective cohort study in Cameroon.

## 2 Methods

### 2.1 Study Design and Setting

We carried out a prospective cohort study in two referral hospitals in Yaoundé, the capital city of Cameroon. These tertiary centers serve as teaching hospitals and have a catchment population of over 2 million inhabitants. The study methodology has been published previously [17]. Patients were recruited between January 2013 and December 2013 and followed up for 1 year (till December 2014).

### 2.2 Study Participants

All consecutive patients with a diagnosis of stroke were seen for possible inclusion in the study. Verbal consent was obtained from patients or a surrogate, and the telephone numbers of those who agreed to be included were obtained as approved by the ethics committee. The data collected were part of routine clinical care in this cohort. We applied the World Health Organization (WHO) definition of stroke as “rapidly developing clinical signs of focal (or global) disturbance of cerebral function, with symptoms lasting 24 h or longer or leading to death, with no apparent cause other than of vascular origin” [18].

### 2.3 Variables

The interview was used to obtain participants' age, sex, marital status, profession, level of education, and residence. Information relating to stroke onset, pre‐existing risk factors including hypertension, diabetes mellitus, dyslipidemia, atrial fibrillation, previous stroke, and heart failure. Hypertension, diabetes mellitus, smoking, ischemic heart disease (with/without ECG changes), alcohol consumption, and history of heart failure were based on history from patients or surrogates. This information was supplemented from data obtained from hospital records. Dyslipidemia and atrial fibrillation were based on a blood test and ECG done during admission.

The initial neurological assessment was performed within 24 h of admission. Stroke severity was assessed using the National Institute of Health stroke scale (NIHSS) on admission [19]. The modified Rankin scale score was assessed on admission and during follow‐up [20]. Routine laboratory and imaging workup served as the basis for stroke etiology and risk factors investigation.

Stroke outcome was assessed starting in the hospital for in‐hospital complications and discharge status. Patients surviving index hospitalization were systematically assessed at 1, 3, 6, 9, and 12 months post‐stroke during hospital post‐stroke follow‐up appointment visits or by phone interview.

### 2.4 Ethical Considerations

The study was approved by the hospital authorities of the Yaounde Central and Yaounde General hospitals acting as the local ethics committee (IRB). The study was performed in accordance with the declarations of Helsinki. We report this study following the preferred standard in reporting observational studies in epidemiology (STROBE).

### 2.5 Statistical Analysis

Data were analyzed with IBM‐SPSS version 26. We compared men and women. Categorical variables are presented as frequencies and percentages with their 95% confidence interval (95% CI). Continuous variables are presented as means and standard deviations (SD). Differences in the categorical variables were assessed with the *χ* ^2^ test or Fishers' exact test where appropriate. The differences between the continuous variables were assessed with the *t*‐test. The main outcome variable was death within 12 months of follow‐up. In bivariate analysis, we assessed risk factors of death and presented the data as Relative Risk with the 95% CI. Factors with a *p* value < 0.2 were considered in the binary logistic regression analysis. In males, the factors considered were age, heart rate, Glasgow coma score (GCS) on admission, NIHSS score on admission, history of stroke, atrial fibrillation, temperature on admission, and the Modified Rankin Scale (mRS) on admission. In females, the factors considered were temperature, GCS, NIHSS score, mRS score, blood pressure, heart rate, and history of stroke. For time‐to‐event outcomes, Kaplan–Meier estimates are presented with the log‐rank test used for comparison between males and females. A *p* value < 0.05 was considered as threshold for statistical significance.

## 3 Results

### 3.1 General Characteristics of Participants by Gender

**Figure 1:**
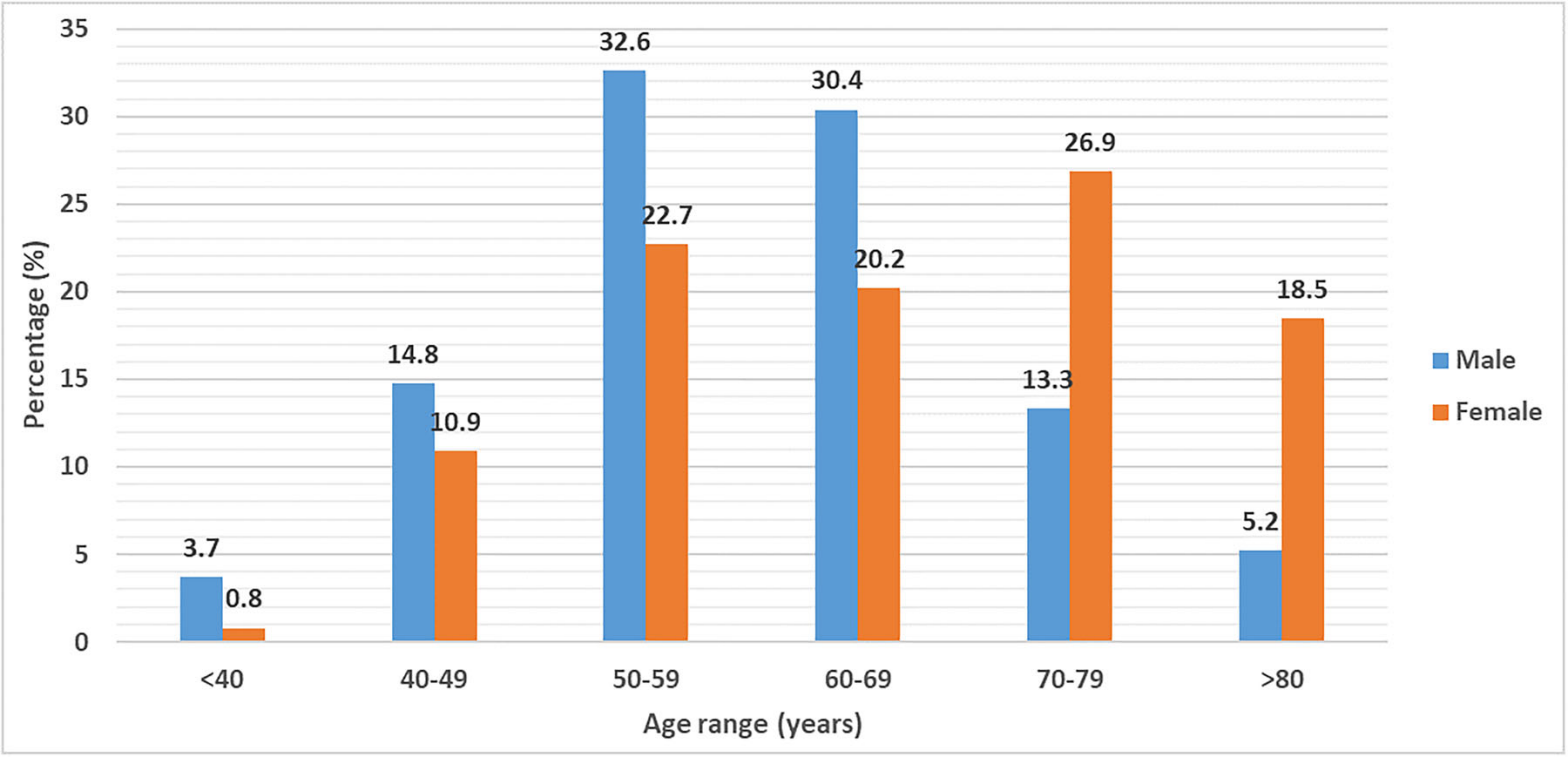
Age distribution by gender.

Table 1 shows the general characteristics of the cohort according to gender. A total of 254 patients with stroke were included in this study at baseline, of which 135 (53.2%) were men and 119 (46.9%) were women. Their mean age was 62.3 ± 15.5 years and ranged from 10 to 95 years. Women were significantly older than men (65.7 ± 15.5 years vs. 59.2 ± 12.4, *p* < 0.001). Figure 1 shows the distribution of age groups according to sex. The most common age group in men was 50–59 years (32.6%), and the most common age group in women was 70–79 years (26.9%). The most frequent cardiovascular risk factors were history of hypertension in 173 (68.1%), diabetes in 50 (19.7%), alcohol consumption in 66 (26%), and tobacco use in 44 (17.3%) patients. Of the 44 participants who admitted using tobacco, 28 (63.6%) were active smokers. The proportion of smoking and alcohol consumption was significantly higher in males compared with females (all *p* < 0.001).

**Table 1:** General characteristics of the participants by gender.

Variables	Overall, (*n* = 254)	Male, (*n* = 135)	Female, (*n* = 119)	*p* value
Age, mean (SD)	62.3 (15.5)	59.2 (12.4)	65.7 (13.9)	< 0.001
Length of hospitalization, mean (SD)	11.8 (8.9)	11.7 (8.3)	12 (9.6)	0.56
History				
Hypertension, *n* (%)	173 (68.1)	90 (66.7)	83 (69.8)	0.599
Diabetes, *n* (%)	50 (19.7)	25 (18.5)	25 (21)	0.618
Previous Stroke, *n* (%)	38 (15)	20 (14.8)	18 (15.1)	0.945
Heart Failure, *n* (%)	13 (5.1)	7 (5.2)	6 (5)	0.959
Atrial fibrillation, *n* (%)	12 (4.7)	4 (3)	8 (6.7)	0.159
Alcohol, *n* (%)	66 (26)	54 (40)	12 (10.1)	< 0.001
Ever smoked, *n* (%)	44 (17.3)	43 (31.9)	1 (0.8)	< 0.001
Clinicals on admission and discharge				
Admission NIHSS, mean (SD)	10.5 (6.2)	9.8 (5.8)	11.2 (6.6)	0.137
Discharge NIHSS, mean (SD)	7.8 (5)	7.3 (5)	8.3 (4.9)	0.128
Admission mRS, mean (SD)	3.8 (1)	3.7 (1)	3.9 (0.9)	0.01
Discharge mRS, mean (SD)	3. 6 (1.4)	3.4 (1.5)	3.8 (1.2)	0.223
SBP, mean (SD), mmHg	177.4 (35.2)	177.5 (36.2)	177.4 (34.2)	0.848
DBP, mean (SD,) mmHg	103 (21.6)	103.8 (22.3)	102 (20.8)	0.410
Pulse pressure, mean (SD), mmHg	74.5 (24.3)	73.6 (23.8)	75.4 (25)	0.584
Heart rate, mean (SD), bpm	81.2 (13.9)	82.3 (14.5)	80 (13.1)	0.204
Temperature, mean (SD), °C	37.1 (0.7)	37.2 (0.7)	37.1 (0.6)	0.532
Glasgow score, mean (SD)	14 (2)	14.2 (1.8)	13.9 (2.2)	0.07
CT stroke subtype				
Ischemic, *n* (%)	162 (63.8)	86 (63.7)	76 (63.9)	
Hemorrhagic, *n* (%)	69 (27.2)	37 (27.4)	32 (26.9)	0.992
Undetermined, *n* (%)	23 (9.1)	12 (8.9)	11 (9.2)	
Mortality				
Admission, *n* (%), (*N* = 254)	56 (22.1)	28 (20.7)	28 (23.5)	0.592
1 Month, *n* (%), (*N* = 198)	25 (12.6)	11 (10.3)	14 (15.4)	0.281
3 Months, *n* (%), (*N* = 173)	9 (5.2)	3 (3.1)	6 (7.8)	0.189
6 Months, *n* (%), (*N* = 164)	13 (7.9)	8 (8.6)	5 (7)	0.714
9 Months, *n* (%), (*N* = 151)	8 (5.3)	5 (5.9)	3 (4.6)	1
12 Months, *n* (%), (*n* = 143)	2 (1.4)	2 (2.5)	0 (0)	NA
Overall mortality in 12 months, *n* (%),	113 (44.5)	57 (42.2)	56 (47.1)	0.439

Abbreviations: DBP, diastolic blood pressure; mRS, modified Rankin scale; NIHSS, National Institute of Health stroke scale; SBP, systolic blood pressure; SD, standard deviation.

On admission, the severity of stroke (NIHSS score) was minor in 46 (18.1%, 95% CI: 13.6–23.4), moderate in 147 (57.9%, 95% CI: 51.5–64), moderate‐to‐severe in 46 (18.1%, 95% CI: 13.6–23.4), and severe in 14 (5.5%, 95% CI: 3.1–9.1) patients. Only one (0.4%, 95% CI: 0.01–2.2) patient had no stroke symptoms. The severity of stroke on admission was similar between men and women (9.8 vs. 11.2, respectively, *p* = 0.137). Women had significantly higher disability on admission compared to men (3.7 vs. 3.9; *p* = 0.01). Hemodynamic parameters, temperature, and Glasgow Coma Score (GCS) were not different between the sexes. Brain computed tomographic (CT) scan was not carried out in 23 (9.1%) patients. Of those with brain CT, 162 (70.1%) had ischemic stroke and 69 (29.9%) had hemorrhagic stroke. There was no significant difference in stroke subtype between men and women.

### 3.2 Outcome

**Figure 2:**
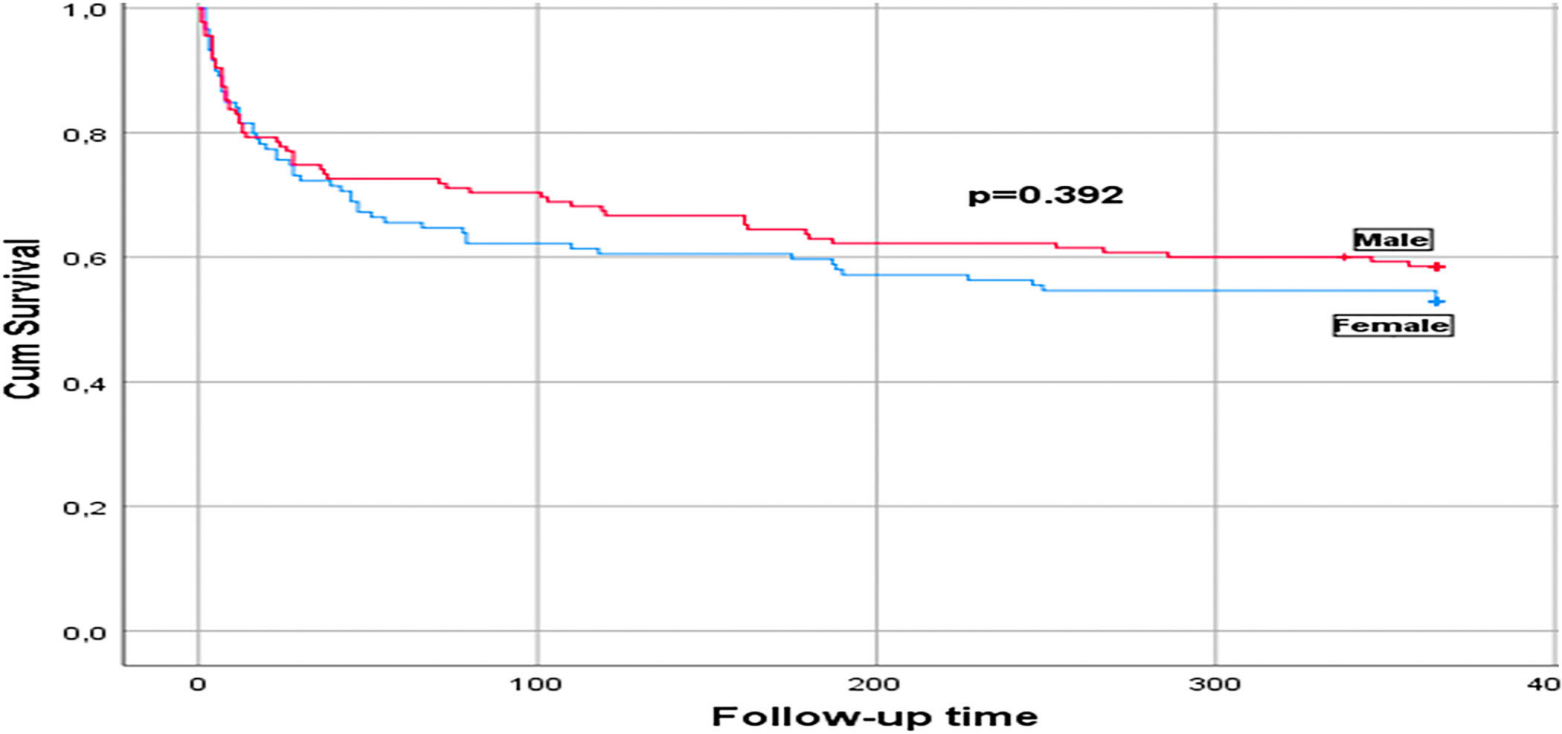
Kaplan–Meier 12‐month‐survival curves post stroke according to gender.

The mean duration of follow‐up was 232.4 ± 161 days, and this was similar between the genders (*p* = 0.566). The follow‐up time was available for all the patients. A total of 113 (44.5%) deaths were recorded, of which 81 (71.7%) occurred within the first month of stroke (Figure 2). The mortality during 1‐year follow‐up was similar between men and women (*p* = 0.329). In bivariate analysis (Table 2), factors associated with an increased risk of death in men were age ≥ 60 years (RR: 1.55, 95% CI: 1.1–2.2), heart rate on admission > 90 bpm (RR: 1.47, 95% CI: 1–2.2), GCS on admission < 14 (RR: 3.2, 95% CI: 1.4–7), and NIHSS score on admission > 15 (RR: 2.4, 95% CI: 1.3–4.6). Factors associated with an increased risk of death in women were fever on admission (RR: 2.63, 95% CI: 1.1–6.3), GCS on admission < 14 (RR: 2.53, 95% CI: 1.4–4.7), NIHSS score on admission > 15 (RR: 2.4, 95% CI: 1.3–4.3), and modified Rankin Score (mRS) on admission > 3 (RR: 1.65, 95% CI: 1.2–2.2). The factors that were independently associated with mortality in men (Table 3) were age [1.05 (1.01–1.09)] and GCS on admission [0.6 (0.4–0.8)]. For every increase in age by 1‐year, the odds of death increased by 5% in males. For every increase in the GCS, the odds of death decreased by 40%. Factors that were independently associated with death in females were the NIHSS score on admission [1.14 (1.02–1.3)]. For every one‐unit rise in the NIHSS score, the odds of death increased by 14%.

**Table 2:** Factors associated with mortality within 1 year of stroke according to gender.

Variable	1‐Year mortality in males			1‐Year mortality in females		
	*N* (%)	RR (95% CI)	*p* value	*N* (%)	RR (95% CI)	*p* value
Age ≥ 60 years						
Yes	34 (55.7)	1.55 (1.1–2.2)	0.004	35 (46.7)	0.98 (0.7–1.4)	0.9
No	23 (31.1)	1		21 (47.7)	1	
Past stroke						
Yes	12 (60)	1.52 (0.9–2.7)	0.08	11 (61.1)	1.42 (0.8–2.6)	0.195
No	45 (39.1)	1		45 (44.6)	1	
Hypertension						
Yes	40 (44.4)	1.12 (0.8–1.5)	0.46	42 (50.6)	1.24 (0.9–1.7)	0.24
No	17 (37.8)	1		14 (38.9)	1	
Diabetes						
Yes	9 (36)	0.9 (0.6–1.2)	0.485	13 (52)	1.13 (0.7–1.8)	0.578
No	48 (43.6)	1		43 (45.7)	1	
Ever smoked						
Yes	18 (41.9)	0.99 (0.7–1.4)	0.953	1 (100)	NA	NA
No	39 (42.2)	1		0		
Alcohol consumption						
Yes	21 (38.9)	0.91 (0.7–1.2)	0.522	5 (41.7)	0.9 (0.5–1.5)	0.693
No	36 (44.4)	1		51 (47.7)	1	
Atrial fibrillation						
Yes	3 (75)	2.35 (0.4–12.9)	0.178	4 (50)	1.1 (0.5–2.2)	0.862
No	54 (41.2)	1		52 (46.9)	1	
Heart failure						
Yes	4 (57.1)	1.37 (0.6–3.3)	0.455	3 (50)	1.1 (0.5–2.4)	0.882
No	53 (41.4)	1		53 (46.9)	1	
SBP ≥ 180 mmHg						
Yes	29 (43.3)	1.04 (0.8–1.4)	0.804	29 (53.7)	1.26 (0.9–1.8)	0.186
No	28 (41.2)	1		27 (41.5)	1	
DBP ≥ 110 mmHg						
Yes	23 (41.1)	0.97 (0.7–1.3)	0.9	21 (60)	1.46 (0.9–1.8)	0.068
No	34 (43)	1		35 (41.7)	1	
Pulse pressure ≥ 65 mmHg						
Yes	37 (45.1)	1.13 (0.9–1.5)	0.396	33 (44)	0.9 (0.6–1.2)	0.382
No	20 (37.7)	1		23 (52.3)	1	
Heart rate > 90 bpm						
Yes	20 (57.1)	1.47 (1–2.2)	0.037	14 (63.6)	1.56 (0.9–2.7)	0.085
No	37 (37)	1		42 (43.3)	1	
Fever on admission						
Yes	16 (57.1)	1.44 (0.9–2.3)	0.073	14 (77.8)	2.63 (1.1–6.3)	0.005
No	41 (38.3)	1		42 (41.6)	1	
Admission GCS < 14						
Yes	19 (79.2)	3.2 (1.4–7)	< 0.001	24 (75)	2.53 (1.4–4.7)	< 0.001
No	38 (34.2)	1		32 (36.8)	1	
Admission NIHSS > 15						
Yes	19 (73.1)	2.4 (1.3–4.6)	< 0.001	25 (73.5)	2.4 (1.3–4.3)	< 0.001
No	38 (34.9)	1		31 (36.5)	1	
Admission mRS > 3						
Yes	48 (45.3)	1.26 (0.9–1.7)	0.169	52 (52)	1.65 (1.2–2.2)	0.02
No	9 (31)	1		4 (21.1)	1	
Hemorrhagic stroke						
Yes	23 (46.9)	1.14 (0.8–1.6)	0.402	24 (55.8)	1.31 (0.9–1.9)	0.15
No	34 (39.5)	1		32 (42.5)	1	

Abbreviations: DBP, diastolic blood pressure; GCS, Glasgow coma score; mRS, modified Rankin scale; NIHSS, National Institute of Health stroke scale; SBP, systolic blood pressure.

**Table 3:** Factors independently associated with death in multivariate analysis.

Variable	Male		Female	
	aRR (95%CI)	*p* value	aRR (95% CI)	*p* value
Age (years) per year increase	1.05 (1.01–1.09)	0.015	NA	NA
Past stroke (yes)	0.4 (0.1–1.2)	0.096	0.6 (0.2–1.9)	0.372
Heart rate (bpm) per beat increase	1.01 (0.98–1.04)	0.52	1.02 (0.99–1.1)	0.233
Temperature (°C) per degree rise	1.58 (0.8–3.1)	0.176	1.33 (0.6–2.8)	0.452
Admission GCS per unit increase	0.6 (0.4–0.8)	0.003	0.9 (0.6–1.2)	0.361
Admission NIHSS per unit rise	1.07 (0.96–1.2)	0.246	1.14 (1.02–1.3)	0.020
Admission mRS per unit rise	0.8 (0.5–1.4)	0.428	1.07 (0.5–2.1)	0.841
Systolic BP (mmHg) per unit rise	NA	NA	1 (0.98–1.02)	0.985
Diastolic BP (mmHg) per unit rise	NA	NA	1.01 (0.99–1.05)	0.347
Hemorrhagic Stroke (yes)	NA	NA	0.98 (0.4–2.5)	0.941

Abbreviations: DBP, diastolic blood pressure; GCS, Glasgow coma score; mRS, modified Rankin scale; NIHSS, National Institute of Health stroke scale; SBP, systolic blood pressure.

## 4 Discussion

This study aimed to examine sex differences in stroke risk factors, clinical presentation and 1‐year outcome in a prospective follow‐up cohort in Yaounde, Cameroon. Our results showed that stroke was more common in men, women were significantly older, and had a greater disability on admission compared to men. Men had a significantly higher prevalence of smoking and alcohol consumption. There was no significant sex difference in the 1‐year mortality during follow‐up. Independent predictors of mortality in men were age and GCS on admission, while the predictor of mortality in women was NIHSS on admission.

To the best of our knowledge, this is the first study to examine sex differences in stroke in a prospective 1‐year follow‐up study in Cameroon. In this study, the proportion of men with stroke was higher compared to women (53.2% vs. 46.9%). This was in accordance with results from a systematic review, which showed that stroke was more common in men than women [21]. A previous study from Cameroon equally showed a predominance of males among admitted stroke patients [14], which may contribute to the perception that stroke is a man's disease. However, the difference tends to decrease with increasing age [22]. Although age‐specific stroke incidence rates are higher in men than in women, stroke affects a greater number of women because of their increased life expectancy and the fact that stroke event rates increase significantly in the oldest age groups [15].

Women with stroke in our study were significantly older than men. This is consistent with findings from previous studies that have reported that women, on average, are older than men when they have their first stroke [23, 24]. In a systematic review by Appelros et al. the mean age at first‐ever stroke was 68.6 years among men, and 72.9 years among women [21]. These ages were, however, higher than the mean age of 59.1 years for men and 65.7 years for women in our study. The mean age in the present study was similar to that reported in a previous study in Cameroon, with mean ages of 58.4 and 62.3 years for men and women, respectively [14]. Stroke occurs in women at an older age. This probably largely reflects sex differences in demographics. Indeed, female sex is associated with a longer life expectancy than male sex, women constitute a larger proportion of the elderly population in which the prevalence of cardiovascular disease in general and stroke in particular is known to be greatest [2, 15].

The sex differences regarding traditional stroke risk factors have also been reported in previous studies. Recent studies have reported that women are more likely to have hypertension, diabetes mellitus, atrial fibrillation, and obesity, whereas men are more likely to have a history of heart disease, myocardial infarction, peripheral arterial disease, current smoking, and alcohol consumption [11, 15, 25]. In the present study, smoking and alcohol consumption, which are lifestyle risk factors, were significantly more prevalent in men. A previous study in Cameroon also showed that men were more likely to smoke and consume alcohol [14]. However, the prevalence of atrial fibrillation, hypertension, and diabetes was higher in women, although the differences did not reach statistical significance. An increased prevalence of atrial fibrillation in women has consistently been reported [9, 26, 27]. Atrial fibrillation is more prevalent among elderly people, and female stroke patients are older when they have their first stroke [10].

In the present study, there was no significant difference in stroke severity on admission between men and women, even though women had a higher stroke severity as evaluated by the NIHSS. The data on sex differences in stroke severity are contradictory. Several studies have shown that women have more severe strokes than men [10, 21, 24, 28]. However, other studies did not report any significant difference in stroke severity between men and women [6, 14, 26].

Data vary as to whether there is a difference in stroke disability between men and women. In our study, compared to men, women had a significantly higher disability on admission. Nevertheless, stroke disability was similar between men and women at discharge. Other studies also reported that women have a worse functional outcome after stroke than men [6, 24, 29]. Some of the factors that have been associated with stroke disability in the literature were previous stroke and age [30]. But a previous study in Cameroon did not report any sex difference in disability on admission [14].

There was no difference in stroke subtypes between men and women in the present study. A systematic review showed that the incidence rates of brain infarction and intracerebral hemorrhage were higher among men, whereas the rate of subarachnoidal hemorrhage was higher among women, although this difference was not statistically significant [21].

Globally, more women die of stroke than men [21]. In the present study 1‐year case‐fatality was higher in women compared to men, although the difference did not reach statistical significance, after adjustment for the aforementioned covariates. Women's all‐cause mortality has been reported to be lower after adjustments for relevant risk factors in some reports [31, 32] but also, conversely, to be higher [21, 33] or similar between men and women [34, 35, 36, 37], as reported in the present study. A similar study in Cameroon examining gender differences in stroke did not report any differences in case fatality [14]. The findings in the study highlight the need for women to receive better stroke care in our setting because they have unique risk factors, and the risk of mortality is higher in women.

### 4.1 Limitation

Our study is limited by the relatively small sample size and the fact that it was conducted in referral hospitals, which may indicate that our findings may not reflect what happens in primary health care services. This must be weighed against the absence of computerized tomography to ascertain stroke diagnosis in primary health care facilities, which usually refer all suspected cases of stroke. Brain magnetic resonance imaging (MRI) with diffusion‐weighted MRI (DWI), which has the most sensitivity and specificity and is the best option to diagnose acute stroke, was not available in the city of Yaounde at the time of the study. Also, our study provided no information on differences in the occurrence of the subtypes of ischemic stroke, such as large artery atherosclerosis, cardioembolism, and small vessel occlusion (lacunar infarction), which may influence stroke severity and disability and outcomes. This study was carried out in the capital city of Cameroon, thus the findings cannot be generalized to the entire country. Despite this limitation, our study is the first in Cameroon to examine gender differences in stroke in a prospective 1‐year follow‐up study.

## 5 Conclusion

As the population ages, the incidence rate and mortality rate of stroke in women will continue to increase. It is imperative to address many of the aspects of stroke presentation, treatment, and care that may differ in men and women. Attention to these differences will ensure equivalent levels of prevention, acute treatment, and diagnostic testing in men and women. Women are more likely to be disabled after a stroke than men. The current literature indicates that this disparity may result from the older age and poorer pre‐stroke functional status of women than men at the time of stroke onset; however, even in age‐matched cohorts, overall functional status is worse in women than men. Differences in hormone exposure, social networks, and comorbid function may contribute to these sex differences in functional recovery. Differences in sex‐based biology are also increasingly recognized. Different strategies (e.g., social interaction, treatment of depression) may be more efficacious in women, and rehabilitation strategies may need to be tailored to the specific needs of women post‐stroke.

## Author Contributions

**Clovis Nkoke:** conceptualization, writing – original draft, methodology, formal analysis, writing – review and editing, investigation, and data curation. **Ahmadou Musa Jingi:** conceptualization, writing – original draft, methodology, formal analysis, data curation, writing – review and editing. **Jean Jacques Noubiap:** conceptualization, formal analysis, writing – review and editing. **Cyrille Nkouonlack:** writing – review and editing. **Anastase Dzudie:** supervision, conceptualization, writing – review and editing.

## Ethics Statement

The study was approved by the hospital authorities of the Yaounde Central and Yaounde General hospitals, acting as the local ethics committee (IRB). All patients provided informed consent before their enrollment in the study.

## Consent

The authors have nothing to report.

## Conflicts of Interest

The authors declare no conflicts of interest.

## Transparency Statement

The lead author Clovis Nkoke affirms that this manuscript is an honest, accurate, and transparent account of the study being reported; that no important aspects of the study have been omitted; and that any discrepancies from the study as planned (and, if relevant, registered) have been explained.

## Data Availability

The data sets used and/or analyzed during the current study are available from the corresponding author on reasonable request.
